# Association Between Cognitive Function and Early Life Experiences in Patients with Alcohol Use Disorder

**DOI:** 10.3389/fpsyt.2020.00792

**Published:** 2020-08-13

**Authors:** Fangshuo Cheng, Shu Cui, Chao Zhang, Ling Zhang, Lei Wang, Qiuyu Yuan, Cui Huang, Kai Zhang, Xiaoqin Zhou

**Affiliations:** ^1^ Department of Psychiatry, Chaohu Hospital, Anhui Medical University, Hefei, China; ^2^ Anhui Psychiatric Center, Anhui Medical University, Hefei, China; ^3^ Department of Pediatrics, Fuyang People's Hospital, Fuyang, China

**Keywords:** early life experiences, attachment, cognitive function, alcohol use disorder, social cognitive function

## Abstract

**Introduction:**

Early life experiences could be potential risk factors for the development of alcohol use disorder. In similar circumstances, it might also influence cognitive impairment in later life. However, the relationship between early life experience and cognitive function in people with alcohol use disorders is unclear. The current study examined the effects of early social environments and experiences on cognitive function in patients with alcohol use disorder.

**Methods:**

A total of 37 adult male patients with alcohol use disorder and 30 healthy control (HC) subjects were enrolled. The MATRICS Cognitive Consensus Battery (MCCB) was used to evaluate cognitive function. The Childhood Trauma Questionnaire (CTQ) and the Revised Adult Attachment Scale (RAAS) were used to evaluate early life experiences of the participants. The RAAS was used to evaluate the attachment patterns of participants.

**Results:**

Compared with the HC group, the alcohol use disorder group reported higher levels of childhood abuse and were more likely to form an insecure attachment style. Patients with alcohol use disorder who experienced trauma performed worse in terms of discrete cognitive parameters such as social cognition, reasoning and problem solving compared to patients without trauma. Importantly, emotional neglect and social comfort were significantly associated with individual social cognitive skills.

**Conclusions:**

Our results suggest that the cognitive function of patients with alcohol use disorder, especially social cognitive function, is affected by early life experiences.

## Introduction

The consumption of alcohol has increased substantially in recent years in developing countries (1). Long-term and excessive consumption of alcohol increases the risk of alcohol use disorder (AUD). Current estimates show that 4% of China’s total population has AUD (2, 3). AUD is associated with various debilitating physical functions, including neurophysiological and cognitive changes (4–6), which can have lasting negative effects on cognitive function even when drinking alcohol infrequently (5). The pathogenesis of AUD is complex, and its occurrence and development may be the result of the interaction between environmental effects and cognitive dysfunction (7, 8). which may lead to a worse prognosis for AUD (9, 10).

It is well known that the exposure to adversity in early life causes a series of negative consequences in later life. Exposure to adversity and poor attachment patterns in early life may lead to negative emotions, and drinkers may use alcohol to ease these negative feelings (11). Extreme stress in childhood may have a serious impact on brain development (12). Neuroimaging studies have shown that repeated exposure to childhood adversity affects the development of the cortical thickness and its surface area (13). Moreover, experiences of abuse in infancy or childhood can have a negative impact on the development of cognitive function in later years (14).

In the 1950s, John Bowlby, a British psychologist and founder of attachment theory, first proposed that attachment is generally defined as a special emotional relationship between a baby and its caregivers. In the process of growing up, the lack of interaction with the object of attachment (such as parents or stable guardians) will have disastrous consequences for a person’s life. In addition to childhood maltreatment, a number of intra-familial influences, such as early separation with caregiver and insecure attachment patterns have also been associated with the development of cognitive function in later life (15).

Early experiences are associated with cognitive development in later life. Cognitive dysfunction may be associated with development of AUD (8). Research on exploring the relationship between social cognition and child trauma is generally conducted in other groups, not in patients with AUD. For example, some studies have examined the relationship between childhood trauma and cognitive function in clinical high-risk mental illness (16). In contrast, others have studied the relationship between childhood adversity and cognitive function in schizophrenia spectrum disorders (17). Some studies found that physical trauma in childhood is negatively associated with the development of working memory and executive function in patients with a psychotic disorder (18–21). The relationship between childhood trauma and cognition among patients with AUD remains unexplored, which is important for their treatment outcome. Hence, in this study, we aimed to investigate the early life experiences, attachment patterns, and their association with cognitive function among individuals with AUD. We investigated the correlations between childhood trauma, attachment styles, social cognition in patients with AUD, and then investigated the predictors of social cognition in patients with AUD.

## Materials and Methods

### Participants

We recruited 37 adult male patients with AUD and 30 healthy controls (HC; all men), with an average age of 36.7 years. Patients were recruited from the Substance Dependence Treatment Center of Anhui Mental Health Center in China between January and June 2019. All HC individuals were selected from the patient caregiver cohort and conformed to the demographic characteristics.

Patients who met the following criteria participated in further testing: 1) confirmation of DSM-IV diagnosis of AUD, assessed by two trained psychiatrists independently using the Structured Clinical Interview for DSM-IV (AUD); 2) aged 18–60 years and Han Chinese to reduce any age related variance in cognitive capacity assessment and bias in the recall of childhood traumas; 3) with a course of disease greater than one year; 4) treated with a benzodiazepine during the acute detoxification period, with more than two weeks of withdrawal time and without any obvious withdrawal response; 5) were able to submit written informed consent and participate in psychopathological measurement. Moreover, we excluded patients satisfying any of the following criteria: 1) comorbid with lifelong psychiatric symptoms, substance abuse other than alcohol; 2) combined use of psychoactive drugs other than alcohol in the past few weeks; 3) abnormal baseline EEG and positive blood screening for benzodiazepines and history of head injury or loss of consciousness in the past 12 months. Furthermore, all individuals’ demographic characteristics were investigated through the questionnaire including age, sex, race, and year of education of parents. Finally, we obtained a complete medical history from all participants, as well as the results of a physical examination. All participants signed an informed consent form. All surveyors had passed consistency training.

### Measures

#### Childhood Trauma Questionnaire (CTQ)

The CTQ is a retrospective questionnaire used to assess adverse events in early life and comprises 28 items (22). Each item is scored on a 5-point-scale ranging from 1–5 (never true =1 to very often true =5). The total score of the CTQ ranges from 25–125. It assesses five types of trauma: physical abuse (PA), physical neglect (PN), emotional abuse (EA), emotional neglect (EN), and sexual abuse (SA). Using the cutoff point (physical abuse ≥ 10; physical neglect ≥ 10; emotional abuse ≥ 13; emotional neglect ≥ 15; sexual abuse ≥ 8), individuals can be divided into two subtypes: maltreated or not maltreated. The CTQ has been reported to have adequate validity and reliability in China (23). Cronbach’s alpha for the factors related to each trauma type ranged from 0.75 to 0.89 in this study.

#### Revised Adult Attachment Scale (RAAS)

The early social environment and social activity were assessed using the RAAS (24), which is an instrument used to assess the attachment pattern of participants. It consists of 18 items, and is divided into three subscales. First, the Closeness subscale consists of six items: 1, 6, 8, 12, 13, and 17. This subscale evaluates the comfort feelings of individuals in close and intimate relationships. The Dependence subscale contains items 2, 5, 7, 14, 16, and 18. This measured the personal internalized feeling that they could rely on others to be available and that others would be in proximity when needed. The Anxiety subscale comprised of six items: 3, 4, 9, 10, 11, and 15, which assess the levels of personal anxiety from interpersonal rejection by others. Each item is rated on a 5-point scale ranging from 1 (strongly disagree) to 5 (strongly agree). Attachment security was defined as a combined score of <36 on the attachment avoidance measures (Closeness and Dependence scales) and <18 on the attachment anxiety measures (Anxiety scale). Attachment insecurity was defined as a combined score of >36 on the attachment avoidance measures and/or >18 on the attachment anxiety measures. The coefficient alphas in this study’s sample were 0.77 and 0.83 for attachment avoidance and anxiety. The RAAS has demonstrated adequate validity and reliability in China (25).

#### Neurocognitive Function Assessments

Cognitive performance in participants was assessed using the MATRICS Cognitive Consensus Battery (MCCB). The battery assesses seven cognitive domains through ten subtests. Speed of Processing tested by Trail Making Test Part A, Symbol coding and Category fluency; Working Memory tested by Wechsler Memory Scale Spatial span and Digital sequence test; Verbal Learning tested by Hopkins Verbal Learning Test Revised; Reasoning and Problem Solving tested by Neuropsychological Assessment Battery: Mazes; Attention/Vigilance tested by the Continuous Performance Test: Identical Pairs. Social Cognition tested by Mayer-Salovey-Caruso Emotional Intelligence Test (MSCEIT): managing emotion. The MCCB battery was translated into a Chinese version, and clinical validity and test-retest reliability were established in the Chinese population (26).

#### Alcohol Use Disorders Identification Test(AUDIT)

AUDIT was used to identify harmful drinkers and chronic alcohol addicts. It contains ten items: the first three items measure the amount and frequency of regular and irregular alcohol consumption; the middle three items measure the symptoms of chronic alcohol dependence; and the last four items assess short-term and long-term problems related to alcohol consumption. 16 ≤ AUDIT < 20 is harmful to use; 20 ≤ AUDIT < 40 is alcohol dependence. The Chinese version of the AUDIT has good reliability and validity among Chinese students (27).

All neuropsychological assessments were performed in the morning with starting times between 9 am and 6 pm. Tests wer performed by trained psychologists who had received consistency training. Participants with AUD were assessed when they were clinically stable.

### Statistical Analysis

In our study, all data analyses were performed using IBM SPSS 22.0. The data were expressed as mean ± standard deviation. We compared differences between the AUD and HC groups using an independent t-test, nonparametric test, and χ^2^ test for continuous and categorical variables, respectively in the first step. The patients were then divided into maltreated versus non-maltreated, and the distinct variables of cognitive function domains and attachment dimensions were assessed between the groups. Next, bivariate Pearson correlation coefficients were used to explore the relationship between CTQ subtypes and attachment dimensions and cognitive function domain scores conferred. The variables displaying significant correlations within multiple linear regression analysis were tested to determine whether adult attachment and childhood trauma were significant predictors of cognitive function. All tests were 2-tailed, and a *p-*value of less than 0.05 was considered statistically significant.

## Results

### Demographic and Clinical Parameters

A total of 51 patients were recruited in the current study; only 37 patients (age = 36.7 ± 8.5 years) met the inclusion criteria. There was no significant difference in the average age and education years (participants and their parents) between patients and HCs. All the selected AUD patients were treated with benzodiazepines during the acute detoxification period, drug withdrawal had reached more than two weeks, and there was no obvious abstinence reaction.

### Early Life Experiences and Attachment Patterns of AUDs and HCs

A total of 56.8% of AUDs reported having experienced at least one childhood trauma. Physical neglect was most frequently reported (45.9%), followed by emotional neglect (35.1%), physical abuse (16.2%), emotional abuse (10.8%), and sexual abuse (10.8%). The frequency of physical abuse and emotional neglect in patients were higher than in HCs. AUDs presented higher CTQ emotional abuse, emotional neglect and sexual abuse estimates than HCs (8.14 ± 2.62 vs. 6.57 ± 1.92, 10.73 ± 5.36 vs. 8.57 ± 2.61, 5.78 ± 1.06 vs. 5.30 ± 0.70, *p <*0.05).

A total of 35.3% of AUDs demonstrated a secure attachment pattern, which was lower than that of HCs (64.7%). The levels of the Closeness and Dependence scales in patients were inferior to HC (19.97 ± 2.40 vs. 22.43 ± 2.43, 13.68 ± 2.26 vs. 18.37 ± 3.16, *p* < 0.05), and the levels of the Anxiety scale were higher among patients than the controls (17.05 ± 3.36 vs. 14.50 ± 5.19, *p* < 0.05) (**Table 1**).

**Table 1:** Comparison between patients with alcohol use disorders and healthy control groups.

Demographics		AUD(N=37, Mean ± SD)	HC(N=30, Mean ± SD)	*t/z/χ^2^*	*p*
	Age (years)	36.7 ± 8.5	35.0 ± 8.7	0.81	0.42
	Education (years)	11.76 ± 2.93	12.33 ± 3.22	0.42	0.75
AUDIT		25.24 ± 5.98	6.60 ± 5.13	13.51	< 0.001
CTQ					
	Childhood trauma,n (%)	21(56.8%)	12(40.0%)	1.86	0.22
	Emotional abuse score	8.14 ± 2.62	6.57 ± 1.92	1.47	< 0.01
	Emotional abuse, n (%)	4(10.8%)	0(0%)	3.44	0.12
	Physical abuse score	7.03 ± 2.54	5.80 ± 1.22	0.94	0.09
	Physical abuse, n (%)	6(16.2%)	0%	5.34	0.02
	Sexual abuse score	5.78 ± 1.06	5.30 ± 0.70	1.17	0.02
	Sexual abuse, n (%)	4(10.8%)	1(3.3%)	1.34	0.37
	Physical neglect score	9.62 ± 2.84	8.53 ± 2.61	1.61	0.11
	Physical neglect, n (%)	17(45.9%)	12(40.0%)	0.24	0.80
	Emotional neglect score	10.73 ± 5.36	8.57 ± 2.61	1.43	0.01
	Emotional neglect, n (%)	13(35.1%)	0(0%)	13.08	< 0.001
RAAS					
	Close scale score	19.97 ± 2.40	22.43 ± 2.43	-4.15	< 0.001
	Dependence scale	13.68 ± 2.26	18.31 ± 3.16	2.45	< 0.001
	Anxiety scale score	17.05 ± 3.36	14.50 ± 5.19	1.68	< 0.01
	Attachment, n (%)	12(35.3%)	22(64.7%)	11.08	< 0.01

Continuous variables are expressed as mean ± standard deviation (SD). AUDIT, Alcohol Use Disorders Identification Test; CTQ, Childhood Trauma Questionnaire; RAAS, Revised Adult Attachment Scale; AUD, patients with alcohol use disorders; HC, healthy control.

### Relationship Between Early Life Experiences and Cognitive Function of AUDs

AUDs with a history of maltreatment were found to perform poorly in reasoning and problem solving and in social cognition than the AUDs with no history of maltreatment. At the same time, the AUDIT score in AUDs with a history of maltreatment was relatively higher (*p* < 0.05) (**Table 2**). Further, while exploring the relationship between childhood trauma, attachment patterns, and cognitive function in patients, the bivariate Spearman’s correlation coefficient was utilized. The scores of physical neglect, emotional neglect, and close attachment were highly correlated with social cognition (r = −0.33, r = 0.52, r = −0.33, *p* < 0.05), while the correlation between the AUDIT score and social cognition was not significant (**Table 3**). The data of physical neglect, emotional neglect, and close attachment were analyzed by stepwise linear regression analyses to explore the association between social cognition (**Table 4**). The regression equation was significant (F_[1,35]_ = 7.02, *p* < 0.05 with adjR^2^ = 0.30). Stepwise linear regression analysis showed that emotional neglect (beta = −0.92; t = −3.12, *p* < 0.05) and close attachment (beta = 1.48; t = 2.24, *p* < 0.05) were significant predictors of variance in social cognition.

**Table 2:** Description of cognitive function measured in patients with alcohol use disorders.

	Non-maltreated(N=16, Mean ± SD)	Maltreated(N=21, Mean ± SD)	*z*	*p*
Speed of processing	32.75 ± 14.71	25.71 ± 10.87	0.88	0.29
Attention/vigilance	39.81 ± 10.42	36.24 ± 11.32	0.69	0.57
Working memory	34.19 ± 8.59	31.95 ± 5.45	0.61	0.63
Verbal learning	41.56 ± 6.89	44.00 ± 11.21	0.82	0.36
Visual learning	39.25 ± 12.62	36.14 ± 13.62	0.74	0.50
Reasoning and problem solving	31.81 ± 4.83	27.67 ± 4.39	1.39	0.02
Social cognition	40.13 ± 10.66	30.71 ± 9.49	1.87	< 0.01
AUDIT	22.63 ± 5.64	27.24 ± 5.55	-2.04	0.04

Continuous variables are expressed as mean ± standard deviation (SD). AUDIT, Alcohol Use Disorders Identification Test.

**Table 3:** Correlations between childhood trauma and attachment styles and social cognition of patients with alcohol use disorders.

	EA	PA	SA	PN	EN	Closeness Scale	Dependence Scale	Anxiety Scale	AUDIT
Social cognition	-0.19	-0.23	-0.16	-0.33^*^	-0.52^*^	0.33^*^	0.26	-0.02	-0.17
Reasoning and problem solving	-0.25	-0.21	-0.29	-0.29	-0.17	-0.22	-0.20	-0.02	-0.08

EA, emotional neglect; PA, physical abuse; SA, sexual abuse; PN, physical neglect; EN, emotional neglect; AUDIT, Alcohol Use Disorders Identification Test.

^*^p < 0.05.

**Table 4:** Predictors of social cognition in patients with alcohol use disorders.

Model	Unstandardized Coefficients		Standardized Coefficients	*t*	*p*
	*β*	*s.e*	*Beta*		
Constant	15.18	13.47		1.13	0.26
Emotional Neglectt	-0.92	0.29	-0.45	-3.12	0.004
Closeness Scale	1.48	0.66	0.32	2.24	0.032

R^2^ = 0.54, adjR^2 ^= 0.30, F_(1,35)_ = 7.02 (p < 0.05).

## Discussion

This study aimed to explore the influence of the early living environment on the cognitive function of individuals with AUDS. The results showed that individuals with AUD were more likely to experience severe trauma at an early age and had more unsafe attachment patterns. Individuals with a history of emotional neglect in childhood perform poorly in social cognitive function, and a safe attachment model has a positive effect on social cognitive function. This may be illustrated by the link between early life experiences and cognitive function; that is, adverse experiences can lead to negative outcomes in adulthood.

Our study found that 56.8% of patients with AUD in rural areas of China reported exposure to at least one childhood trauma, and physical neglect was most frequently reported (45.9%), followed by emotional neglect (35.1%). This is inconsistent with the findings in developed areas (28); low-or-middle-income countries presented higher estimates of childhood neglect than the high-income countries (29). Patients with AUD with severe childhood trauma experiences presented poorer skills in reasoning, problem solving, and social cognitive functions. One study reported that individuals exposed to physical and emotional events in the initial stages of their life may have impaired verbal skills and social cognitive functions (17). A history of emotional neglect is an independent risk factor for AUD (28). Our findings demonstrated that exposure to emotional neglect in childhood has a negative association with social cognition function performance, as evidenced in the MCCB test in individuals with AUD. Deficits in social cognitive function are hallmarks of major psychiatric disorders, causing weakened social competence, impaired community functioning, and deterioration in quality of life (30, 31). Relevant studies have investigated the impact of childhood trauma on social cognitive function (32–34). Our findings concur with the findings of Kilian, Asmal (17) that emotional neglect is negatively associated with social cognition within MCCB. The effects of emotional neglect are transmitted based on the common environmental factors that arise from the parents and family environments (35). Children with experiences of emotional disturbances have a higher propensity to develop sensitivity toward social cues, which may cause failure to perceive the social information (36). Across different contexts (37, 38), it has been indicated that individuals, both in HC and psychiatric disorder patients, who are victims of emotional trauma, display worse performance in tasks involving emotional perception, theory of mind decoding, and social inference. Moreover, the experience of emotional neglect in early life (0−6 years) could contribute to deficits in theory of mind (39). Heleniak and McLaughlin (40) also reported that violence would alter the cognitive and affective theory of mind.

AUDs with characteristics of insecure attachment (41). Our study found that a higher level of closeness to an attachment figure was strongly associated with increased social cognitive function in patients with AUD. The structure of attachment formed in initial life could have a top-down effect on social information processing. Exposure to appropriate maternal mind-mindedness is beneficial for the evolution of infants’ social understanding (42). Individuals with insecure attachment patterns tend to show lower levels of understanding of social information and social cognitive skills than those with secure attachment patterns (30). For example, anxious attachment is associated with a deficit in social cognitive function, and avoidant attachment showed a U-shaped association with social cognitive, individuals with medium scores on avoidant attachment displayed worse theory of mind than lower and higher individuals (43). Further, the exposure to insecure attachments in early life may interrupt the interaction with caregivers, decrease social interaction exposure throughout life, lead to a lack of adequate experiences to communicate an understanding of mental states during early development, and impair the development of the hippocampal neurons, resulting in a decline in cognitive function (44, 45).

It has been found that exposure to stress is negatively associated with long-term hypothalamus-pituitary-adrenal axis activity leading to glucocorticoid release and activation of the ascending dopamine system as well as impairment of specific brain regions (e.g., hippocampus and prefrontal cortex, major areas involved in learning, memory, and social information processing) (46). The stress also has regulatory effect on the function of neuropeptide. For example, Wagner and Echterhoff (47) et al. reported that the impact of oxytocin on memory accuracy relies on individuals’ attachment patterns. It is positively affected in individuals who find dependence on others uncomfortable and negatively affected in individuals who find dependence on others comfortable. Additionally, Cancel, Comte (48) et al. found that the stability of connectivity among brain regions involved in emotional processing such as the amygdala and the precuneus, the posterior cingulate cortices, and calcarine sulcus, are affected by a history of childhood abuse. Moreover van Schie, van Harmelen (49) et al. also mentioned in the context of emotional trauma, the ability to understand the mental states of others is related to increased activation of the left inferior frontal gyrus.

Exposure to adversity in early life has a lasting effect on the development of brain function. Individuals with impaired cognitive function, especially impairment in social cognitive, had a poorer quality of life than the HCs (31). Furthermore, such patients are more vulnerable to psychological and physical violence (50). Any kind of misery (viz. domestic violence, childhood abuse and neglect, and economic adversity) contributes to the deterioration in quality of life and are potential risk factors for the onset of early drinking problems (51). Individuals exposed to trauma in early life may adopt the use of alcohol to deal with trauma-related symptoms (52). In the context of stress, addicts are more likely to increase their alcohol consumption, which could further lead to more trauma exposure. Meanwhile, the effect of trauma might be transferred across generations and negatively affects an infant’s cognitive and neurologic development. Molenaar, Tiemeier (53) et al. revealed that maternal psychopathology and stress during pregnancy are associated with elevated cortisone in the hair of the offspring, which might affect the function of the cortex. Thus, the transmission of unsolved traumatic experiences could be intergenerational (54). Therefore, protecting patients who are vulnerable to exposure to violence and helping post-traumatic patients make them aware of the impact of trauma to avoid being affected by violence is a necessary practice in clinical intervention.

In our results, patients with AUD drink more alcohol, have more social cognitive impairment and more childhood maltreatment. Therefore, is the social cognitive impairment of people with AUD caused by alcohol use or childhood maltreatment? Our results show that, among AUDs, people with childhood maltreatment have more cognitive impairment than those without childhood maltreatment. In the correlation analysis, there was no significant correlation between social cognitive impairment and AUDIT score, while the correlation between social cognitive impairment and childhood maltreatment was significant. Our results are supported by previous research reports (55–58). One study found that, in men, there were no differences in cognitive decline among alcohol abstainers, quitters, and light or moderate alcohol drinkers (< 20 g/d) (55). A two-year longitudinal study that enrolled 12,408 participants by Stampfer et al. (56) showed that moderate drinkers had better mean cognitive scores than nondrinkers; up to one drink per day does not impair cognitive function and may actually decrease the risk of cognitive decline. A prospective cohort study (57) found that alcohol consumption was U-shaped in all areas of cognitive function, and low to moderate alcohol consumption was associated with a better overall cognitive score.

There are several limitations to the current study. First, participants had diffused and unclear memories of the early adverse experiences, which could introduce a risk of recall bias. Second, when we designed the experiment, we did not collect the subjects’ alcohol use characteristics and other general demographic information. Third, the study used cross-sectional data and small samples, excluding any causal inference. Moreover, in the present study, no female patients were enrolled. Loi, Mossink (59) et al. confirmed that females may be more resilient to early life stress. Further studies are required to investigate the relationship between early life experiences and social cognitive abilities, focusing on sex-based analyses.

## Conclusion

The main findings of the current study include an enhanced understanding of the relationship between early social environment, early life adversity, and cognitive function in individuals with AUD. In particular, the association of emotional neglect and attachment figures could be significant predictors for development of social cognitive function. Therefore, early intervention in trauma, good parent-child relationships, and modification in the deficit internal working models of social interaction are immensely important to influence the recovery of cognitive functions and psychological adjustment.

## Data Availability Statement

The raw data supporting the conclusions of this article will be made available by the authors, without undue reservation.

## Ethics Statement

The studies involving human participants were reviewed and approved by Ethics Committee of Chaohu Hospital, Anhui Medical University (No. 201901-kyxm-02). The patients/participants provided their written informed consent to participate in this study.

## Author Contributions

FC and SC contributed equally to this article. (I) Conception and design: XZ. (II) Administrative support: XZ, KZ. (III) Provision of study materials or patients: FC, SC, LZ, CZ, QY, CH, LW. (IV) Collection and assembly of data: FC, SC, LZ, CZ. (V) Data analysis and interpretation: FC, CS, KZ. (VI) Manuscript writing: All authors. (VII) Final approval of manuscript: All authors.

## Funding

We thank all of patients who volunteered to participate in the study. This study was supported by the National Natural Science Foundation of China (81801341), the Anhui Provincial Key R&D Programme (202004j07020030). These funds are only used to provide a small amount of financial compensation for each research participant participating in the research.

## Conflict of Interest

The authors declare that the research was conducted in the absence of any commercial or financial relationships that could be construed as a potential conflict of interest.

## Abbreviations

AUD, alcohol use disorder; HC, healthy control; MCCB, MATRICS Cognitive Consensus Battery; CTQ, Childhood Trauma Questionnaire; RAAS, The Revised Adult Attachment Scale; PA, physical abuse; PN, physical neglect; EA, emotional abuse; EN, emotional neglect; SA, sexual abuse
